# The Influence of Climate and Other Environments on the Distribution and Character of Disease*Read at the Thirty-first Annual Meeting United States Veterinary Medical Association, September, 1894.

**Published:** 1894-10

**Authors:** W. L. Williams


### THE INFLUENCE OF CLIMATE AND OTHER ENVIRONMENTS ON THE DISTRIBUTION AND CHARACTER OF DISEASE.*

By W. L. Williams, V. S.

The influence of climate, housing, food, care and other surroundings induce marked changes upon the distribution and character of disease, whether among animals or mankind; and the student of pathology needs to weigh these forces carefully, that he may avoid error by arriving at conclusions based upon observations confined to a limited area.

Both in those diseases known to be transmissible and in those supposed not to be capable of passing from one animal to another we find many instances that the disease is narrowly confined to a limited area, and never seen outside of it. Others are usually confined to comparatively limited areas, passing rarely and spasmodically beyond their usual boundaries, soon to retreat therein again; others exist mainly in certain centers, with isolated cases disseminated over a large portion of, or the entire animal world; while others know no boundary, apparently, except that of animal life: all presenting interesting variations in character, the reasons for some of which we can know or estimate.

---

*Read at the Thirty-first Annual Meeting United States Veterinary Medical Association, September, 1894.

The study of the various influences controlling the distribution and character of disease belongs largely to the domain of philosophical medicine, and offers for consideration many highly interesting and instructive problems, but it is no less a part of practical medicine, giving new insight into many of these peculiar epizoötics, as well as to a large number of extraordinary cases of common affections, which at times so baffle and worry the practitioner. The extraordinary case in one locality, under one class of conditions, frequently constitutes the ordinary disease under wholly different surroundings.

The study enlightens us too as to the etiology of many diseases, since we not infrequently find the prevalence of a disease in a certain locality attributed to certain well marked local environments, when in fact the same disease, and of like character, exists in regions where the supposed essential etiological factor of the former locality is wholly absent. So it is that we find our standard writers attributing a disease at times to some local cause, when a more general knowledge of the distribution of the disease would show them that it existed under conditions requiring some other explanation for its existence than that given.

Again, we find our writers describing symptoms of a disease as those characteristic of a malady, when in reality they are overlooking the essential characters, and are portraying vividly what they have seen in a limited area, perhaps in compass of a few square miles in a great city.

It is not deemed desirable to undertake the consideration of a great number of diseases, but rather a few important affections, which are more or less prevalent in the United States, which it is believed will serve as a ground work for an interesting discussion.

In order to secure as complete data as possible in the brief time at my disposal, letters were addressed to about one hundred of the leading veterinarians of this country, almost all to members of this Association, and representing, as nearly as possible, all sections of the United States, asking for their observations as to the influence of climate and other environments upon the distribution, character, course and termination of the following diseases: Glanders, tuberculosis, actinomycosis, bursattee, enzoötic spasm of the larynx, poll evil and fistulous withers, osteo-porosis, rhachitis, periodic or specific ophthalmia.

The responses to these letters were unexpectedly numerous, and were very gratifying in the amount of interest manifested, and the amount of valuable data contributed, and I wish to acknowledge here, with sincere gratitude, what I have not been able to do individually, my obligations to my colleagues for their kind assistance in the preparation of this paper.

It will be noted that some of the diseases very markedly limited by geographical lines have not been considered in our list, although their behavior offers some very interesting points for study.

We are all very well aware of the closely defined area in which Texas fever is confined as an indigenous disease, its boundary being fixed fundamentally, to all appearance, by thermal conditions. The so-called loco disease of the dry regions of the West seems to be coexistent with the prevalence of the loco weeds, the *oxytiopis Lamberti* and closely allied plants, although up to the present the relationship between the plant and the disease has not been defined.

Other diseases of a local character, such as "mountain fever" and "joke easy," or "kidney disease," prevalent in certain Rocky Mountain regions, which have been but indifferently or not at all described, depend apparently upon local conditions, the nature of which are at present not even surmised.

Other diseases depend for their distribution and character upon the density of animal kind, like hog cholera and bovine pleura pneumonia, and tend to pass over infected countries in rhythmic waves, the lowest point of prevalence and mortality following closely the decrease in numbers of animals, and the acquired immunity to a severe pestilence, to be again followed when the general conditions of the country are favorable to disseminations; animal numbers have increased and the disease in some focus has found favorable conditions for luxurious development, when the disease again spreads over a large area as a great epizoötic.

The distribution and character of other diseases are largely determined by the prevailing use to which animals are put, thus infectious abortion of cows predominates in dairying regions and far less frequently in localities devoted to the breeding of beef cattle. Again other diseases may exist without prejudice to the native animal in a country where it is indigenous, and become a virulent pestilence once it oversteps its indigenous territory; or susceptible animals are imported into its territory, like Texas fever and rinderpest.

Coming to the brief list of diseases we have selected to specially consider, first, in order of arrangement, is glanders, one of the longest known, most carefully studied and most widely disseminated diseases of animals; running an indefinite, erratic course, assuming multitudinous forms, and offering, on the whole, one of our most interesting subjects from the standpoint now viewed. Glanders among solipeds is more or less prevalent in every country so far as known save Australia, irrespective of temperature, humidity, altitude or other climatic conditions. It attacks its victim in spite of food, housing, care, age or breed, yet its prevalence, transmissibility, character, duration are all as susceptible to the influence of such as is the most delicate barometer to changes in atmospheric pressure, and becomes so changed in aspect that there is no resemblance in any way save bacteriologically between a series of cases.

Fleming (1) notes that the disease is highly prevalent in Continental Europe and northern and eastern China, rare in South Africa, unknown in India except as an imported affection, and coming as a rule from Australia where the existence of the disease is denied. He also quotes Liquistin as stating that the disease was unknown in Mexico until introduced by the U. S. troops during the war with the country in 1847, but that failed to spread to any notable extent.

Prof. W. Williams (2) states that glanders is a disease of temperate climates, diminishing in prevalence in hot or cold countries.

Robertson (3) agrees substantially with Williams, adding two exceptions to the general rarity of glanders outside temperate climates by noting its prevalence in Norway and Java.

Friedberger and Fröhner (4), citing Krabbe, state that by statistics collected in 1857, 1873, that for each 100,000 horses there were annually found glandered in Norway, 6; in Denmark, 8.5; in Great Britain, 14; in Sweden, 57; in Wurtemburg, 77; in Prussia, 78; in Servia, 95; in Belgium, 138; in France, 1130; and in Algiers, 1548.

In the State of Illinois from 1886–1892 inclusive there were reported annually 13 glandered horses for each 100,000. Other figures of value in the U. S. were not available, and the prevalence of the disease can only be considered comparatively.

In the New England States, which are cool and not moist, the disease seems to be rare, and to pursue a mild, chronic course gen-

(1) Veterinary Science and Police.
(2) Principle and Practice Veterinary Medicine.
(3) Equine Medicine.
(4) Special Pathology and Therapeutics.

erally. In Maine, according to the cattle commissioner's report, there were destroyed in 1891 18 glandered animals, and in 1890 the same number. They claim, however, that a large percentage of cases were traceable directly to importation from Boston, Mass.

Dr. Choate (Lewiston, Me.) reports glanders of a mild, chronic type fairly common in his vicinity. Dr. Peters (Boston, Mass.) reports the disease rare. While Dr. Paige (Amherst, Mass.) reports it rare except in large cities.

Judging from the report of the Massachusetts Cattle Commissioners for 1887, glanders in a very mild, chronic form was quite prevalent among the street-car horses of Boston.

In a special report by Dr. Winchester it seems that 192 animals out of 1700 were quarantined as diseased or suspicious, out of which but 4 animals were killed, although Dr. Liautard had positively pronounced 31 glandered and 28 suspicious. Of the 192 animals quarantined 20 were released by Dr. Winchester's motion, while 162 were released by the Board over Dr. Winchester's protest, although this latter number included nearly all those positively condemned by Drs. Liautard and Huidekoper. It seems that acute, well defined cases were almost unknown in this outbreak, and that the quarantining of the affected and suspected animals by Dr. Winchester served evidently, as Dr. Huidekoper notes in his report of inspection, to ameliorate the symptoms, under the beneficent influence of rest, good food and improved hygienic conditions. There was a strong tendency, it seems, in these cases to spontaneous recovery, and it is well to note here that close quarantine, i. e., the withdrawal from labor, with good food and care, always tends in all localities to remission in symptoms and a tendency to recovery, either apparent or real, almost always the former, thus tending to bring the official veterinarian into disrepute, and by bringing about many apparent recoveries tends strongly to domicile and perpetuate the malady.

In New York and Brooklyn, according to Dr. Burns (Brooklyn), the disease is comparatively rare at present, doubtless due more to the influence of well enforced sanitary police regulations, than to climatic influences, the latter being quite favorable to the spread of glanders in a virulent form.

In Pennsylvania, Dr. Pearson (Phila.) reports the disease on the increase, but leaves us in the dark as to the type assumed; it is reported comparatively rare in Ohio by Dr. Howe (Dayton), and its prevalence at a ratio of 13 in 100,000 horses in Illinois, has already been noted.

Climatically, New York, Pennsylvania, Ohio, Indiana and Illinois may be grouped together as comparatively humid States, with sudden and great thermal variations, but generally neither excessively hot nor cold. The climate does not call for very warm stables, rendering the question of overcrowding and bad ventilation a minor one, and being highly productive and prosperous agricultural region, most horses are well fed and receive careful handling. The unfavorable climatic conditions are counterbalanced largely by favorable surroundings in other respects. In this series of States we find every possible type of glanders, the chronic prevailing, although the acute is not uncommon, mainly in the cities, and due evidently to overcrowded, unsanitary stables. In country districts, quite notably in central Illinois, many cases exhibit a mild type, with very distinct tendency to spontaneous recovery.

Animals, which from their history have evidently been affected for six or eight years, have at no time been incapacitated in the least for ordinary work, but have in every way, except occasional cough and slight nasal discharge, with slight tumefaction of submaxillary lymphatics and characteristic ulcerations, or cicatrices, given all evidence of robust health. Others have come under our observation where an entire stable of horses had become affected apparently with glanders, all alike in an acute form, with emaciation and profuse nasal discharge, some of which entirely recovered; others partially recovered and after two or three years were condemned and killed, while the recovered animals exhibited no signs of disease, although remaining in close contact with the animals plainly affected. Acute cases, apparently approaching rapidly a fatal termination, frequently turn under favorable conditions and make very marked improvement.

The laws for killing prevent opportunities for very extended observations.

In New York, Dr. Law (Ithaca) observed to me in a private conversation, that he had seen at Ithaca permanent recovery of the disease.

Wisconsin, Michigan and Minnesota probably differ but little from the series of States just considered. Dr. J. S. Butler (Minneapolis) noted that in his experience the disease is more prevalent and more acute in large cities, where horses are crowded in ill ventilated stables. He also remarks that in his locality, where the winters are cold, rather uniform and dry, the disease apparently remains latent in winter and develops in spring. This observation applies certainly, with some variations, to much of the territory considered, but not so evidently as Dr. Butler has observed, since in the more southerly States just considered the entire winter may be, and not infrequently is, almost throughout, very like the spring of Minnesota, cool, wet and variable. Hence, often the winter season is the period for greatest development in these localities, while a dry summer, with abundant food, may operate favorably.

In Missouri, Kansas and Arkansas horses, except in cities, are very little stabled, the climate being milder and no marked tendency to humidity, which condition seems to very markedly affect the type of glanders, the disease being quite thoroughly disseminated, but generally causing no great loss, spreading very slowly and attacking but few animals in a band. Dr. T. J. Turner (State Veterinarian, Columbia, Missouri) observes that, while widely disseminated, the disease is but feebly communicable, and assumes almost constantly the mild type in outdoor animals, and the acute type in those closely stabled. Dr. White (Sedalia, Mo.) reports the disease almost constantly mild and very feebly transmissible. Dr. Dinwiddie (Fayetteville, Arkansas) reports glanders in mules as generally a mild, chronic course, in direct contradiction to the generally accepted ideas of the disease in this animal in all parts of the world.

Dr. Mayo (Manhattan, Kansas) notes that the nasal discharge in glanders is less in his region, he thinks owing to a dryer atmosphere, than observed in more northerly and easterly States.

Dr. G. A. Johnson (Sioux City, Iowa) has observed the disease almost wholly in the chronic form, but notes no recoveries. Dr. W. B. Niles (Ames, Iowa) has noted the disease in the mild form, and during dry seasons has noted apparent recoveries, leading him to believe that under favorable conditions spontaneous recovery is quite possible.

In the South Atlantic and eastern Gulf States we meet with a warmer and more humid climate, solipeds generally are of less value, mules largely predominate, and as a rule they are not so well fed and cared for as in other sections considered.

Dr. Kilborne (Washington, D. C.) has found the disease quite prevalent in the District of Columbia, Maryland and Virginia, usually in a mild, chronic form. Under rigid sanitary laws the disease has decreased in the District of Columbia, from 78 cases in 1889 to one case every month or two at present. He has noted one case of spontaneous recovery from nasal glanders, two from farcy, all proven cases of glanders, and the recovery for one year after disease seemed in every way complete.

Dr. Cary (Auburn, Alabama), comparing glanders in Alabama with his observations in Iowa and Illinois, finds the disease more virulent in the former State, owing to increased humidity, with heat.

Dr. W. B. Niles (Ames, Iowa), who has observed the disease in South Carolina and Iowa, finds that in mules in South Carolina it runs an acute course, while in horses he notes no difference in South Carolina and Iowa.

Dr. Tait Butler (Agricultural College, Miss.) reports the disease rare in his State. Prof. A. W. Biting (Florida) reports that in Florida isolated cases are rare, and acute in form. The diseased animals, if not already in their ownership, are soon transferred to negroes, who are usually poor care takers.

In humid regions along the Pacific Coast the disease is frequent and virulent.

Aside from some very small areas, we have briefly glanced over the United States, except the Rocky Mountain region and the high and to a great extent arid plains sloping from their base towards the Mississippi River, a large part of which is cold, some of it extremely so, all quite dry, even to converting much of it without irrigation into a great desert, the altitude varying from 2,000 to 8,000 feet, and horses kept generally in large bands, without stabling. They are usually placed in such environments as permit, with labor, the procuring of a liberal, if not abundant, supply of food. In this entire region the disease is very common, mild, but feebly contagious, and tends to recover spontaneously.

Dr. A. H. Baker (Chicago, Illinois), upon a trip to South Dakota in 1885, found glanders very prevalent, and was especially struck with the very mild type it assumed, horses doing their accustomed labor for seven or eight years without apparent detriment, and showing no constitutional defects whatever, yet by local lesions showing plainly and unmistakably that they were affected with glanders.

Dr. Hinebauch (Fargo, N. D.) reports the disease as assuming the same form in his State, and to this mild, largely latent type, rendering detection difficult, he attributes its wide prevalence. Dr. S. Stewart (Kansas City, Kansas), observing glanders along the Missouri River, Dakota to Arkansas, finds it in a very mild form, and has noted several apparent recoveries, either spontaneous or under indifferent treatment. Dr. Waugh (Allegheny, Pa.), observing glanders in California, Arizona, New Mexico, western Texas and along the northern border of old Mexico, finds it prevalent among horses, mules and asses, being most rare in the latter, and assuming a very mild type. The acute form is rarely seen. This mildness Dr. Waugh attributes to the hot, dry climate, alkaline water, and grasses richly impregnated with mineral substances. Treatment appeared favorable, yet he failed to produce recovery in cases tried in New Mexico.

In the San Joaquin Valley of California glanders has recently prevailed very largely, and led Dr. Klench (Am. Vet. Review, Vol. XIII, p. 214) to contribute a very remarkable article upon the disease, under the name of general lymphangitis, giving as faithful description of glanders as can well be found in veterinary literature, in history, symptoms, termination, etc., yet holding, on the most palpably erroneous grounds, that the disease was not glanders. In this locality Dr. Waugh has noted spontaneous recovery in horses.

In Montana horses are ranged on lands 3,000 to 7,000 feet above sea level, have usually abundant food and a dry climate. The past year has been exceptionally wet, and as an immediate result glanders has developed more commonly than before, and in more severe form. Of 40 cases inspected three were acute in character, two common work horses, indifferently cared for, and one a draft stallion, kept stabled.

I had occasion to inspect some 2,000 range horses for glanders, at the annual round up, and while I found on the range 12 glandered animals, all but two of them had worked on valley farms where glanders existed, and where they had either contracted or developed the disease. Of the two unbroken range animals found affected, one was a two-year-old gelding, the other an aged brood mare affected for five years. Both were in first-class general health, and showed but slight local lesions. In most of the animals affected for the longer periods this disease was uniformly of a very mild type. One recent case I have watched for a few months was severely affected constitutionally and locally, in January, this year, but has rapidly improved and is now apparently well, except small cicatrices in nostril, and very slight glandular tumefaction.

The disease in Montana must be said to assume a remarkably mild type, and on the ranges is barely a contagious disease, and although farm animals badly affected are allowed at idle seasons to intermingle freely with range stock, yet the disease is rarely seen among them. Perhaps it is far more common among these range, or practically wild, horses than it would seem, but remains wholly latent, or in a mild internal form, until the animal is caught and subjected to the fatigue of rough breaking, when it develops. It seems highly probable that some range horses acquire the disease in a mild form, recover and thereafter possess immunity.

Post-mortem examinations indicate less pulmonary infection than at lower altitudes.

In England the disease assumes, apparently, a more acute type than in most parts of the United States, and Prof. Williams (Prin. and Practice Vet. Med.) holds that the only practical possibility of recovery is in mild cases of farcy. Fleming (Vet. Sanitary Science and Police, Vol. I, p. 505) admits the possibility of recovery in mild cases of farcy.

In Continental Europe, it would seem, it assumes a milder type than in England. Leading professors, Dickerhoff (1), and Friedberger and Fröhner (2), and numerous others, admit the possibility of occasional recovery, spontaneously, or by the aid of medication.

War has at all times and in all countries proven a prolific cause of glanders; the fatiguing work, the great exposure, frequently in a climate wholly new to the animal, the scanty and damaged food supply, the want of proper care, the intimate, immediate contact of healthy with diseased animals, in close barracks, or at times in still closer transport ships, and the larger number of external wounds, galls and abrasions, rendering superficial inoculation easy, all serve to give the disease great virility, and cause it to spread as a serious scourge. This is not all, for, as Dr. Kilborne observes, regarding prevalence in Virginia, Maryland and elsewhere, is traceable in many of the Northern and border States directly to the purchase of affected horses from the United States Government at the close of the Civil War. The animals which had acute glanders, and would soon have died and ceased to have been a source of danger, were killed, while the dangerous animals, those with mild, chronic glanders, being impaired for military service, were sold in every direction, causing a wide dissemination of the disease.

It has been claimed that breed influences the character and distribution of glanders, but this is probably indirectly. Some say that lymphatic draft horses suffer more severely, but in central

(1) Special Pathology and Therapeutics.
(2) Special Pathology and Therapeutics.

Illinois, much devoted to breeding high class, heavy draft stock, the disease appeared far less, and of milder type, in them than in other breeds. It appeared to be especially the disease of the poor man's horse. The better classes owned the more valuable draft stock, and if one were ailing, the nature of the malady was learned by employing a veterinarian, and proper action taken, or the diseased animal was sold or bartered at a low figure to the poorer neighbor, and the disease, with the aid of unsanitary surroundings, communicated to his inferior animals.

To-day we find glanders most prevalent in those sections of our country where it runs the mildest course, shows the greatest tendency to recover, and is the least contagious. In such localities, at present, we find the most and the cheapest horses. The mild cases are difficult of diagnosis, and stock owners cannot be convinced of the character of the disease.

In the Rocky Mountain region the vast herds of wild horses cannot be satisfactorily inspected, and the mallein test in these is out of the question, so that the mildly diseased animals cannot be detected. Owners have been taught to believe that glanders is uniformly and rapidly fatal, hence take no alarm from a feeble nasal discharge, which disappears at some seasons of the year, the animal continuing in good general health and performing good labor year after year. Many owners are, in their own mind, competent judges of the matter, and relate how much they saw of it during the war, but are not aware that they only saw acute cases, and failed to note the mild cases, which, taken from the Army and sold, scattered seed, the fruit of which we are still harvesting.

Veterinarians, too, with little experience are slow to call glanders in many of these localities by its right name, but, like Dr. Klench in the paper already noted, worry themselves to find some other name by which to hide their error as to its real nature. Our British and American writers and teachers give too vivid a description of the virulence of glanders, and the average graduate locating in the dry regions of the West is illy prepared to condemn a horse for glanders, that has been barely visibly affected for seven or eight years, or one that has been ailing for a few months and is rapidly recovering.

To control glanders, we need to appeal to intelligence, where it exists, and in its absence, to force. When an intelligent horseman has been working a fat, robust, glandered horse for seven or eight years, it does not appeal to his intelligence to say to him that his animal is affected with an unavoidably and rapidly fatal disease.

I once treated a suspected animal for a few weeks, and then casually asked a competent officer to examine the horse and he unhesitatingly pronounced the horse sound, and I concurred, but the horse had glanders, and we both eventually concurred upon that point, but the owner very properly called another veterinarian, because the history of the case, and our opinions, had not appealed strongly to his intelligence.

Again I saw a pony with severe glanderous pneumonia, which an experienced veterinarian said would die in a few days, but when we forcibly killed her some months later she had largely recovered, showed little constitutional signs of the disease.

In another case a veterinary officer condemned a horse severely affected, shot it in the head at close range with a shot gun, saw it roll over and go into its death struggle, when, on account of a storm, he turned away. Later the owners helped the animal to his feet, fed him liberally, the hole in his head healed, the symptoms of glanders almost vanished, and some time later, when the horse had apparently recovered, the veterinarian found it difficult to persuade the owner that his horse was affected with a surely and rapidly fatal disease, and must be killed.

I claim that it is not appealing to intelligence to urge the destruction of glandered horses upon the ground that it is either rapidly or surely fatal, because such assertions are evidently incorrect, and horse-owners can see it as well as veterinarians. Science is truth, and the prevailing notions of the course and termination of glanders are not true, and veterinarians are largely responsible for this condition.

I have been surprised and gratified by the unanimity with which those I have asked to aid me in this paper have, when any opinion at all has been expressed, granted the possibility of recovery in cases of glanders.

In bovine pleura-pneumonia we found ample reasons for quarantine and slaughter, when only a small percentage of cases resulted fatally, and the disease was not committed to man. How much more may we urge slaughter when, eventually, nearly all cases prove fatal, and at all times the danger of human infection confronts us?

Tuberculosis is, in many respects, allied closely to glanders, and its distribution and character are modified by similar influences. In both diseases the course and duration is indefinite; the bacilli, arranging themselves in groups in various tissues and organs, become encapsuled and eventually tend to perish therein.

Both affect largely, both primarily and secondarily, the lungs, and both attack preferably the lungs of those individuals among susceptible species, other environments being equal, in which these organs are not constantly very active, but contain at some, if not at all, times a large amount of residual air. High altitudes with consequent dry atmosphere bring about special chest development, and require for the sustenance of life a much more complete and active respiration, and permit much less residual air. Post-mortem examinations of glandered horses at high altitudes, as before stated, have thus far, in my experience, indicated a much less development of pulmonary tubercles than in lower altitudes, in like severe cases. Close stabling, without exercise, produces results quite parallel to low altitudes with humidity, hence where the two are combined we would expect, and do find, the greatest prevalence and most virulent type of tuberculosis, while in the high altitudes, with dry atmosphere, it is unknown, except as directly imported.

Dr. Waugh (Allegheny, Pa.) saw tuberculosis only in sojourning human invalids in California, New Mexico, Arizona and old Mexico. The same holds true in Montana, and, so far as I can learn, throughout the Rocky Mountain region, although cattle abound everywhere, but are rarely stabled. The only case of tuberculosis reported to me as being originated in the West was one by Dr. A. H. Baker, in a Colorado bullock killed at Chicago stock yards.

As we approach the Mississippi River the disease becomes somewhat prevalent, so that Dr. W. B. Niles (Ames, Iowa) and Dr. D. A. Johnson (Sioux City, Iowa) report the disease somewhat prevalent in that State, especially in stabled herds, and Dr. S. Stewart (Kansas City, Kansas) reports whole herds of cows infected in western Nebraska, and Kansas and eastern Colorado, chiefly in the udder, without general lesions.

The infection in these cases he charged to diseased bulls brought from the East. Dr. Mayo (Manhattan, Kansas) sees the disease only in highly bred cattle, and in Arkansas Dr. Dinwiddie reports it practically unknown, except in highly bred, closely housed herds, which are rare, and in Mexico Dr. S. E. White (Sedalia, Mo ) reports essentially the same condition.

Dr. LeMay (Fort Riley, Kansas) reports no tuberculosis.

Dr. J. S. Butler (Minneapolis, Minn.) reports the disease rare in that State, but observed it frequently in stabled dairy cattle in Ohio. While Dr. Howe (Dayton, Ohio) reports it comparatively rare in Ohio, confined mainly to dairy cattle.

In the Southern States cattle are, as a rule, poorly bred, rarely housed or overfed, and not generally used for dairying purposes, while generally considerable exercise is obligatory in order to obtain food, and large numbers are not brought in close contact with each other. Dr. Tait Butler (Agricultural College, Miss.), Prof. A. W. Bitting (Lake City, Florida), Dr. Cary (Auburn, Alabama), report the disease very rare in Southern States.

As we approach the central Atlantic States where many highly bred cows are kept, closely housed, highly fed, and induced to yield milk to their utmost capacity, we find the disease enormously increased in frequency and virility. Drs. Clement (Baltimore, Md.), Pearson (Philadelphia, Pa.), Kilborne (Washington, D. C.), Paige (Amherst, Mass.), and Peters (Boston, Mass.), all report the disease as highly prevalent among cattle in their regions, ascribing its great prevalence to high breeding, inbreeding, excessive lactation, excessive feeding, close housing, with other unsanitary conditions. Dr. Peters estimates that in New England 1 to 2 per cent. of cattle are tuberculous, while in Eastern Massachusetts there is probably 3 to 5 per cent. affected. Dr. Pearson notes it is increasing in Pennsylvania. Drs. Peters and Pearson note a tendency, in some cases, to recovery with the same suspicion that we all regard this tendency to recovery from glanders. They constitute the greatest danger to the health of other animals.

Dr. Schwarzkopf (Chicago, Ill.) offers some suggestions regarding tuberculosis, which deserve more than a passing notice.

Foremost in the etiology of the disease he places confinement to hot stables during the summer and winter months, which assists infection. "You know," he says, "that dairymen found out that they get more milk by keeping their cows in-doors. Experimental stations have been teaching this, and this point has been overdone. Nothing reduces more the vitality of the animal and the inherent resistance to diseases than lack of exercise. This is one of the reasons why we find so many fat cows tuberculous."

To these deductions I most heartily subscribe, and have especially noted, for a long time, the pernicious teachings of some of our experimental stations founded by our government for the purpose, among other things, of fostering our live stock and dairy interests, which, by this pernicious teaching, they are constantly tending to destroy by destroying the vigor of our cattle.

They teach that a cow yielding 20 or 30 lbs. of butter fat per week is exercised abundantly by secreting milk. These stations are founded for scientific experimentation, but there is no science n such teaching.

We admit that in a short space of time more milk and butter fat can be taken from a cow with a given amount of food, but the process is contrary to all physiological and hygienic laws, and constitutes an enormous drain upon invaluable reserve forces which we can never replace.

Actinomycosis is rarely seen in New England and the Eastern States. Drs. Clement (Baltimore, Md.), Peters (Boston, Mass.), Paige (Amherst, Pa.), Kilborne (Washington, D. C.), Pearson (Philadelphia, Pa.), Choate (Lewiston, Me.), Howe (Dayton, O.), all report it practically unknown except by importation from the Western States.

The disease is rarely met with in the Southern States. Drs. Dinwiddie (Arkansas), Tait Butler (Mississippi), Cary (Alabama), and Prof. Bitting (Florida), having observed the disease very infrequently.

The central Mississippi valley, the West and Northwest appear to be favorite regions for the development of this disease. I have seen it assume an epizoötic form in Illinois during exceptionally dry seasons, attacking sometimes 30 and 40 per cent. of a herd of 20 or 30 animals. Drs. J. S. Butler (Minneapolis, Minn.), T. J. Turner (Missouri), LeMay (Kansas), T. E. White (Missouri), G. A. Johnson (Iowa), S. Stewart (Kansas), W. B. Niles (Iowa), A. H. Baker (Nebraska), and Waugh (California, Arizona, New Mexico and old Mexico), all report it very common, and this seems the general condition all along the Rocky Mountains, and those semi-arid States sloping eastward from their base, except it seems North Dakota, in which Dr. Hinebauch reports it very rare.

In all localities there appears to be a tendency, especially in the external lymphatic type, to spontaneous recovery through suppurative destruction of the affected glands, and the iodide of potash treatment seems successful in a large percentage of cases.

It seems to be attributed, by common consent, in most cases to wounds caused by germ-infected, hard, coarse food, but this seems to account for the means of infection and geographical distribution of the germ itself, a question which at present seems unanswerable. It seems that were the germs equally distributed, sufficient coarse food would be encountered in Eastern States to frequently produce the necessary wounds for infection.

Bursattee has been so largely described as a disease of British India, and so scantily, and that chiefly in periodical literature, that its presence is not generally looked for in this country, and far from all veterinarians have given the matter sufficient notice to recognize a case when seen, and it is feared that the reports so kindly sent me are not wholly reliable as to the geographical distribution of this malignant, infectious disease.

R. W. Burke and other East India writers describe the disease as prevailing during the rainy season, and say it abates or temporarily recovers upon cessation of latter, or upon removal of animals to dry hills. It is scarcely noted elsewhere in the old world

It is reported in the United States by Dr. Waugh in California, Arizona, New and old Mexico, by Dr. Hinebauch (frequently) in North Dakota, by Dr. W. B. Niles in Iowa, by Dr. Stewart in Nebraska and Kansas, by Dr. Mayo in Kansas, by Dr. G. A. Johnson in Iowa, by Dr. LeMay in Kansas, by Dr. J. S. Butler in Minnesota and Ohio, by Dr. C. C. Lyford in Minnesota, by Dr. Tait Butler in Mississippi, by Prof. Bitting in Florida.

It prevails extensively throughout central Illinois. I have seen it in Indiana, and it is met with occasionally in Montana. It rarely assumes a fatal type in the United States, but is persistent. It recovers spontaneously, as a rule, upon the approach of cold weather, to reappear at the same part in worse form upon the return of hot weather. While heat and moisture seem to play an essential part in its prevalence and character in India, heat seems the essential factor here, and determines its existence regardless of humidity, altitude, food, housing, care or other environments. The source of the infecting agent seems wholly unknown.

In not too severe cases it yields fairly well to treatment, when consisting of the destruction of a large part or all the germs containing tissues, followed by desiccating, antiseptic dressings.

The disease I have, for want of a better name, termed enzoötic spasm of the larynx, has been very rarely and indifferently described, and seems from all reports to be rare in the United States.

Veterinarians Joseph Leather & Sons (*) describe an outbreak of this disease under the head of lathyrns, poisoning in the horse, and attribute the disease to the feeding of lathyrns sativa, or Indian vetch. In this outbreak 21 out of 35 horses attacked were destroyed. In the same article reference is made to

---

*Vet. Journal, Vol. XX, p. 233.

cases of the same lathyrns poisoning occurring in the practice of Dr. McCall.

Dr. Gresswell has seen much of the disease in Colorado where the lathyrns sativa is not grown or fed.

Geo. S. Witter (*) describes the disease as it occurred in five of his breeding stallions in Colorado, but cannot suggest the cause.

Recently suit was brought in a Montana court to recover damages from the owner of an ore mill, by a horse breeder who had lost many horses from this affection, and alleged their death was due to poisons in mill tailings, which were scattered over his pastures in irrigating—quite a different cause from that suggested by the Drs. Leather.

Dr. Choate thinks it occurs rarely in Maine, although he has not seen it personally.

Dr. W. B. Niles has not seen the affection in Iowa, but has heard of an enzoötic in that State which is probably this disease.

In 1884 I observed an interesting outbreak of this disease in central Illinois, chiefly in a land of full blood and high grade French draft animals. Winter had set in early and frozen much of the green corn (maize) to such an extent that it was not worth gathering, and the animals were turned into the fields and allowed to eat the frozen ears of corn with which the advent of warmer spells of weather had putrefied.

Four or five animals in this outbreak died, while an equal number recovered. Horses kept in stable on same farm, but fed on selected maize, not frozen, remained healthy, and I was led to attribute the disease to the putrefying maize The animals had no access to lathyrns sativa, or any bean or vetch.

The etiology of this disease seems shrouded in the deepest mystery, and there is nothing positive as to any influence exerted by climate, season, altitude, housing, etc., but probably to some unknown element occurring accidentally, but rarely and locally in food. My own cases and those of Drs. Leather and McCall suggest some form of mould, due, in my case, to frozen, putrefying maize, in the London and Edinburgh outbreaks, possibly to a mould multiplying on the vetches while somewhat damp on shipboard, but the Montana and Colorado cases tend to abrogate this idea, and rather suggest a bacterial contamination of

---

*American Veterinary Review Vol. XII, p. 437.

food, not due to excessive moisture and not confined to grains, since some of these cases occurred on the range, where moisture is not excessive and grains not seen.

FISTULOUS WITHERS AND POLL EVIL.

These two forms of the same disease show marked peculiarities in distribution. It occurs in every part of the United States, regardless of temperature, altitude, humidity, housing, food, breeding or other environments, but it varies greatly in its prevalence and character in different localities, and the belief of veterinarians as to etiology changes with prevalence and location.

In the States east of the Mississippi River the disease is reasonably frequent, but occurs as isolated cases, almost always traceable to traumatism, exhibiting no tendency to spontaneous recovery, but yielding somewhat tardily to surgical treatment.

In the Southern States, east of the Mississippi River, the disease is more common than in the more northerly States, and its increased frequency is variously explained. Some say it is because of the greater number of mules, which have a great tendency to annoy other animals, and especially to bite the withers roughly and persistently. Others attribute its prevalence in a great measure to the carelessness of negro owners in the matter of low stables and ill-fitting harness and saddles.

In the States west of the Mississippi River, with but few exceptions, the disease breaks out frequently in enzoötic form, and at once suggests other causes than traumatism, although these admittedly cause isolated cases here as elsewhere.

Dr. A. H. Baker, of Chicago, viewing the disease from a distance, attributes its wide prevalence in the West to bites and rough handling by other horses when in pasture. Dr. Waugh, of Pennsylvania, observing the disease in the extreme Southwest, attributes it to rolling on stones, due to the torments of insects.

But these reasons do not explain the sudden appearance of the disease in a larger per cent. of horses over a certain area, and its disappearance again in the course of a few years.

In Montana it prevails very extensively now; some cases in unbroken animals on smooth ranges devoid, practically, of serious insect pests, but it is more common in small bands of horses kept in pastures in the valleys, not worked, ridden, or stabled, and having no perceivable reason for suffering from any unusual number of traumatisms; yet it will suddenly appear in two or three, or even six or eight horses in one small band; in some as poll evil, in others as fistulous withers, and not infrequently both appear at once in the same animal, or one may closely follow the other. Both show a marked tendency to spontaneous recovery through suppurative processes. It occurs in the stable too, as well as in the field, and I have seen poll evil and fistulous withers appear simultaneously in isolated horses, kept in good stables, with good care. and no history of wounds.

Its distribution among horses in enzoötic form, so far as we can learn, appears to coincide geographically with actinomycosis of cattle, a fact to which Dr. Reynolds has already drawn attention. A relationship has been suggested on account of their like distribution.

They bear other minor resemblances at times, and we occasionally see in poll evil, or fistulous withers, large, indurated swellings, with small, oft-recurring abscesses, pursuing a very chronic, persistent course.

In cases there appears, too, a well marked tendency to disease of osseous tissue, not typical of ordinary caries. In two cases, without symptoms of caries, I have seen in cases of poll evil ante-posterior perforation of occipital protuberance, with discharge of pus from the poll evil escaping through this bony channel on the forehead. On the whole, poll evil and fistulous withers in enzoötic form, like actinomycosis, seem to be diseases of prairie localities, and do not affect timbered regions. Of all States east of the Mississippi River, Illinois, the chief prairie State, seems the only one in which the prevalence of this disease cannot be accounted for satisfactorily on the traumatism theory. Dr. T. S. Butler, of Minneapolis, draws attention to a notable fact, in that in his region, as in the case in Illinois, the disease is rare in cities in comparison to the number of cases occurring on farms. Were traumatisms the sole cause such should not be the case.

Periodic or specific ophthalmia offers some very interesting facts in its geographical distribution. It seems common in the British Islands and Continental Europe, although not so prevalent as in parts of the United States. In our country the disease occurs frequently in the New England and Atlantic States, due largely to importation from the Mississippi Valley. It appears to arise frequently in the Allegheny Mountains, and is reported common in Maine by Dr. Choate, and in Massachusetts by Dr. Paige.

Along the coast it seems to disappear almost wholly, except as an imported disease, so Drs. Pearson, Peters, Kilborne, and others report it very rare, although other practitioners report a more frequent occurrence.

In Alabama, Dr. Cary reports the affection frequent and acute, but in general the disease seems not very common in the South.

The disease occurs most frequently in the States bordering upon the Ohio River, and upon the Mississippi River above its junction with the Ohio, reaching its greatest prevalence in the United States, and probably in the world, in Illinois, Iowa, and Missouri. In more northerly States it tends to disappear, so that Dr. Lyford, of Minneapolis, sees it largely in imported horses.

As the high, semi arid country east of the Rocky Mountains is, approached the disease becomes far more rare, and less dangerous, yielding readily, in most cases, to care and treatment. So in Nebraska and North Dakota, Drs. Stewart and Hinebauch report it very rare and mild.

Reaching a still greater altitude, with less rainfall and humidity, the disease vanishes as we approach the Rocky Mountains, to reappear again at a few of the lower altitudes along the Pacific Coast.

This disease has no parallel in geographical distribution. It vanishes wholly in those regions where tuberculosis and tetanus cease to exist, but prevails to its greatest extent in regions where the latter are comparatively rare, and in turn becomes rare where tuberculosis and tetanus prevail most frequently and severely.

The etiology has been explained by some teachers and writers. apparently to their entire satisfaction. I was taught that it was largely hereditary, and that the shining example of this was to be found in the progeny of the famous blind sire, Lexington, and so in my early practice I frequently found the cause in a remote infusion of Lexington blood, attributing to other causes the more frequent appearance of the disease in draft stock not related to Lexington. In the Rocky Mountain region we find as much of the Lexington blood, comparatively, as anywhere, the original breeding stock having been derived very largely from Kentucky, and bands of thoroughbreds are found, almost every animal of which may be traced back to the illustrious blind horse, yet I have not seen a horse in the Rocky Mountains affected with periodic ophthalmia, except newly imported animals, and these recover promptly unless very far advanced, and remain permanently free from the disease. My observations in this region have been very brief and limited, but are in full accord with the more extensive experience of Drs. Knowles (Montana), Waugh (California, Arizona, New Mexico, and old Mexico), Turner (Fort Niobrara, Nebraska), and other veterinarians and stock owners of this region. In fact I have been unable to learn of a single case of this disease arising in the entire Rocky Mountain region, whereas, were heredity a sufficient factor in the causation of the disease, the entire region, where carelessness in breeding reaches its highest point, should be thoroughly overrun with this affection.

Inbreeding is mentioned too as a fertile cause, yet in the large bands of wild horses in this region, colts grow up, are left uncastrated, and mate promiscuously with parent or offspring without producing evil results in this respect.

Close, ill-ventilated stables, especially dark stables, are also cited as fruitful causes, but in the northern Rocky Mountains, where low temperature prevails, stables are very dark and unsanitary, yet the few horses kept in them are free from the disease.

Others attribute the disease to the ill effects of bright sunlight, especially to snow glare in the winter season, but this disease is more of a summer affection, and does not occur so largely in winter in any district, so far as observed, and were snow glare an important factor, the Rocky Mountains should be the chief habitude of this disease, since the snow glare of this region is far beyond that of the States where the disease prevails.

No one living in the Mississippi Valley, or eastward, can begin to appreciate the intensity of the snow glare, as seen at high altitude in the Rocky Mountains, with its dry, calm atmosphere, and excessively low temperature during winter.

Others attribute the disease to food, and since the disease reaches its highest prevalence in the chief corn producing States, maize is held largely responsible for its prevalence. There is good reason for believing this food to be an important factor in its cause, but not in itself sufficient to bring it about, or at least it falls far short of being a chief or essential cause.

The disease is not uncommon in many regions in this country where corn is rarely fed, and appears frequently in England and Continental Europe where corn is not fed at all.

Neither is corn fed along the Pacific Coast where the disease prevails. It is hard to determine, under these conditions, if food can ever suffice to produce the disease, or if the great prevalence of the disease in the chief maize territory is a mere coincidence.

That temperature, and more especially altitude and humidity, exert a powerful influence upon the occurrence of this disease there can be no doubt.

Dr. Clement (Baltimore, Md.) suggests yet another element in the causation of this disease, worthy of careful consideration, and in order that I may give you his views clearly I quote his words : " There is an affection, from casual observation, identical with specific ophthalmia, but which is infectious beyond doubt, and which attacks horses without history of what we call specific ophthalmia, sudden attack of conjunctivitis, followed in a few hours with acute iritis and pus in the anterior chamber. Disease amenable to treatment by rest, counter-irritation and atropia injections. If not attended to apt to be followed by adhesion of iris to lens, and sometimes cataract. Either this is different from specific ophthalmia, or we are all wrong in description of latter disease. I am inclined to latter belief."

Dr. T. J. Turner (Columbia, Mo.) gives a description of the disease essentially identical with that quoted from Dr. Clement, and I have noted in severe cases in Illinois the same symptoms, course and termination. I would add also, that on several occasions on farms where the disease had not before occurred, and in families where prior history had been wholly clean in respect to this disease, and where the most careful examination failed to discover any cause in, or even change of food, housing, or care, in any way whatever, that the disease would suddenly appear in several animals in a very virulent form, running an exceedingly acute course, terminating frequently in blindness at the first attack. In this way I have seen four or five animals, out of a band of seven or eight horses on one farm, all previously sound and without hereditary taint, suddenly attacked with virulent ophthalmia, ending in permanent blindness, the disease then vanishing, leaving the sound animals perfectly well. In these cases all parts of the eye appear inflamed, tense and painful, the conjunctivæ hyperaemic, the cornea opaque, and the anterior chamber filled with lymph, pus or blood. Dr. Clement's suggestion of infection in these cases is certainly worthy of careful consideration, as it may possibly explain them, which cannot be satisfactorily done by the theories advanced heretofore.

To say the least our accepted ideas, as to the etiology of periodic ophthalmia, need revision.

